# Clinical exposure is not enough: legitimacy as the missing link in preparing future doctors

**DOI:** 10.3389/fmed.2026.1953149

**Published:** 2026-09-15

**Authors:** Stuart Redvers Pattinson, Hans Savelberg, Anique Atherley

**Affiliations:** 1 Centre for Health Sciences Education, Faculty of Health Sciences, University of the Witwatersrand, Johannesburg, Gauteng, South Africa; 2 School of Health Professions Education (SHE), Maastricht University, Maastricht, Netherlands; 3 Department of Nutrition and Movement Science, School of Health Professions Education (SHE), Faculty of Health, Medicine and Life Sciences, Maastricht University, Maastricht, Netherlands; 4 Director of Medical Education, The University of the West Indies, Cave Hill Campus, Cave Hill, St Michael, Barbados

**Keywords:** communities of practice (CoP), Global South, glocalisation, legitimacy, preparedness for practice

## Abstract

Glocalization is commonly understood as the adaptation of globally endorsed educational ideals to the reality of local contexts. This framing often assumes a unidirectional flow of innovation from the Global North to the Global South. In this perspective, we challenge this assumption through the lens of preparedness for practice. Evidence from the Global North consistently identifies insufficient experience with patient care as a key contributor to graduates' lack of preparedness for clinical work, with an increasing use of tools such as simulation to try fill this gap. In many Global South settings, however, authentic clinical exposure is abundant, presenting an opportunity for the Global South to set the standard regarding the preparation of graduates while the Global North must grapple with local constraints. Yet concerns regarding graduate preparedness persist. Drawing on literature and our research with South African internship doctors, we argue that clinical exposure alone does not guarantee preparedness for practice. Rather, the critical factor is whether participation within clinical environments is legitimized. Students may be surrounded by authentic clinical work while remaining peripheral to it, prioritizing examination performance over meaningful engagement and experiencing exclusion from clinical teams. We propose legitimacy, a sense of belonging and validation within a community of practice, as the key bridge between exposure and preparedness. From a glocalization perspective, this argument highlights the responsibility in the Global South not to just simply implement Global North strategies but rather to identify local opportunities and solutions, and in doing so provide valuable insights that contribute to global discourse about how best to prepare future doctors.

Glocalization, centred on the idea of “thinking globally and acting locally,” (1, 2) aims to balance global standards with the realities of local training environments. Health professions educators strive for curriculum innovation that is connected to pedagogical ideals but recognises the reality of local demands (3), adapting globally endorsed educational models to their unique contexts (4). Implicit in this framing is the assumption that educational innovation flows primarily from the Global North to the Global South, with the latter tasked with contextualising externally developed standards and solutions. The narrative often extols the idea that “west is best,” othering and excluding the Global South's contributions which are seen as only relevant to local audiences (4, 5). Yet this dynamic between the Global North and South does not always necessarily hold true, and there may be cases where contextual limitations in the Global North hinder the enactment of pedagogical ideals. In such situations there is a risk that health professions educators in the Global South, instead of recognising local advantages and an opportunity to lead the way, may feel the pressure to adopt Global North solutions for problems they might not actually have, all in an attempt to meet the status quo. Such assumptions reinforce a subtle but persistent narrative that educational excellence originates in resource-rich settings and is subsequently translated for use elsewhere (4). Yet, educational advantage is not distributed as neatly as material resources. We recognise that neither the Global North nor the Global South represents a homogeneous educational reality. Considerable complexity and variation exist within regions, institutions and health systems. We therefore use these labels heuristically to highlight recurring patterns described in the literature rather than fixed geographical or cultural categories (4). While institutions in the Global North may possess sophisticated simulation centres, extensive faculty development programmes and highly structured curricula, they often struggle to provide students with authentic participation in patient care. Conversely, many Global South settings offer unparalleled opportunities for immersion in real clinical work (5, 6). Glocalization therefore requires more than local adaptation of global ideas; it requires recognising that innovation and educational insight can flow in multiple directions.

An example is preparedness for the transition to practice—the extent to which graduates possess the knowledge, skills, confidence, adaptability and social capabilities to manage the new responsibilities, relationships, and identity changes associated with navigating the shift from student to doctor (7). Literature from the Global North suggest that medical graduates are under-prepared for the transition to practice in a number of important ways (7–15). Graduates struggle with the complex demands of healthcare systems and feel inadequately trained in communication skills, practical “know-how” and the management aspects of the job (15–17). They experience difficulties with taking responsibility and decision making (18, 19), and have been found to be under-prepared for clinical reasoning, navigating conflict and team hierarchy, working effectively in multiprofessional teams, understanding hospital systems, dealing with emergencies, applying knowledge to practice, coping with uncertainty, managing workload and maintaining a work-life balance (8, 13, 18, 20). Dornan et al. aptly describe unpreparedness as “knowing what but not how” (21). The predominant reason given for this under preparedness and lack of confidence among junior doctors is insufficient experience with real-life patient care (22–24). This is understood to be the consequence of rapid patient throughput, rotation based training, increasing outpatient treatment, fewer clinical signs, inconsistent clinical exposure and patient safety concerns and guidelines that limit student's authentic involvement in clinical teams (17, 24–27). This has led to an increased use of tools such as simulation to try fill this gap (27). We should remember, however, that simulation is intended to be an adjunct to, not replace real-world clinical experience (21, 27). Ironically, many of the educational conditions that institutions in the Global North are attempting to recreate through simulation, assistantships, longitudinal placements and entrustment frameworks already exist in many Global South training environments (5, 6, 27). High patient volumes, workforce shortages and service demands often place students *near* authentic clinical work (5). From a glocal perspective, the challenge may not be creating opportunities for clinical exposure, but ensuring that participation—meaningful engagement in the activities, responsibilities and shared goals of a community—is educationally legitimised.

In Global South contexts such as in South Africa, a quadruple burden of disease, infrastructural deficiencies, limited resources and low doctor-patient ratios may create a need for students to be more hands-on and involved in patient care (5). The complex challenges of medical training in South Africa present an apparent opportunity for medical schools in the Global South to be readying more resilient, adaptable and stronger junior doctors, setting the standard in preparedness for the transition to practice (5). There may even be assumptions that the problem of a lack of hands-on experience leading to unpreparedness couldn't possibly be an issue in these settings. Yet, it seems this potential to optimally ready students for work is not always realised (28–30). Our own research with internship doctors in South Africa suggests that medical graduates are entering practice not ready in the ways that count in reality (28–30). We have found that newly qualified junior doctors struggle with the adaptability, efficiency, communication skills, teamwork, practical know-how and confidence needed to manage the demanding workload in a fast-paced, resource constrained environment (28–30). Insufficient exposure does not adequately explain the under-preparedness described by graduates in the South African context. Without the same contextual constraints on students gaining clinical experience, what is hindering the preparedness for practice of students who are being trained in high clinical exposure contexts? If students learn in the very clinical environments that many Global North institutions are attempting to emulate, why do similar concerns regarding preparedness persist? And what can be done to ensure the opportunities of training in such a setting are taken advantage of? These are important questions to answer, as well-prepared graduates result in better patient outcomes as well as improved junior doctor wellbeing and retention (21, 26). The answers may lie in a distinction that is often overlooked in discussions of workplace learning: exposure is not the same as participation. While the term “exposure” is often used to refer to learning in authentic clinical environments, it does not necessarily equate to experience and may imply passivity rather than participation (31). Students can be surrounded by authentic clinical work without being recognised as legitimate contributors to it (30). In this respect, preparedness for practice is not simply a function of how much clinical experience students encounter, but whether their participation within that experience is legitimised.

Legitimacy is a sense of belonging and validation that is derived from being recognised as a valued participant in a community of practice. This is achieved through a process of legitimation; understanding, and then demonstrating, the knowledge, skills and ways of being that are valued in that context, allowing for participation in a way that is rewarded by the community (32). What is legitimised shapes learning; students' behaviour will almost always be guided by what is valued and forms the basis of achievement within the curriculum (17). Communities of Practice (CoP) provides a useful theoretical lens for understanding how legitimising participation within clinical teams can enable students to be better prepared for the transition to practice. According to CoP theory, if novices, such as medical students, start out as legitimate peripheral participants then, through gradually developing a shared repertoire, demonstrating competence and assuming increasing levels of responsibility, they can become more experienced and central members of a community (33). Through opportunities to participate and meaningfully contribute to the community's shared aims, students are encouraged to develop the professional identity, interpersonal skills, clinical competence and understanding of the healthcare system that is required to successfully transition to the workplace (34).

In our research, however, we found that in medical school, instead of participation, it is performance in high stakes, off-the-job assessments of knowledge this is legitimised and forms the basis of achievement. This encourages students to seek out formal teaching and study time over involvement in the clinical space (28–30). Time in short clinical rotations is largely orientated towards exam preparation rather than student participation. Students are often made to feel “in the way” by hierarchical clinical teams, which see students as an added burden in a strained healthcare setting rather than a valuable extra pair of hands. The overall result is that, despite learning in the same demanding setting in which they will later work, students are not gaining the confidence, practical know-how and collaboration skills they need for the transition to practice (15). Clinical exposure is not enough, whether student participation is legitimised within those experiences makes the difference (32, 33, 35, 36).

For the potential of clinical training in the Global South regarding preparing upcoming physicians for the transition to practice to be realised, health professions educators, curriculum designers and faculty leaders need to carefully analyse their campus climates, institutional cultures, assessment practices and curriculum designs, examining what forms the basis of achievement in their settings and how it is communicated, enacted and experienced. If students are excluded or marginalised by clinical teams instead of being recognised as legitimate participants, then their confidence and engagement will be undermined and their professional identity formation (progress towards thinking, acting, speaking and feeling like a doctor) will be impeded (15, 37). If preparing for assessments and preparing for the transition to internship are seen as competing priorities, opportunities for students to gain relevant experience will be missed. As long as high-stakes theory assessments are privileged over the development of personal and social skills, graduates will remain underprepared for the realities of practice (17). When students experience a sense of belonging in clinical teams and are given meaningful roles, they are motivated to learn (17). Providing students with graded responsibility during longitudinal clinical placements allows for trusting working relationships to develop and can provide students with an understanding of the responsibilities and demands associated with the strained system in which they will later be working, allowing them to regulate their own learning towards being prepared for the role (17, 21). Patient safety remains a priority, but as Lefroy et al. suggest; withholding authentic real-time tasks from students “risks more than it resolves with respect to patient safety” (24). Proposed solutions to patient safety concerns, such as the use of simulation (27), can still be of great value in the Global South, but not as a substitution for authentic involvement in patient care but rather as a means to equip students early on in their studies with the skills they need to safely get more hands on as senior students.

Preparedness for practice illustrates an important limitation of how glocalization is often conceptualised within health professions education. Too often, glocalization is framed as the adaptation of Global North innovations to Global South realities. Yet, the challenge described in this paper demonstrates that while educational problems may be shared their underlying mechanisms differ substantially across contexts. The preparedness challenges described in many Global North settings are frequently attributed to limited opportunities for authentic clinical participation (17, 24). In response, educators and institutions have invested heavily in simulation, assistantships and clinical models designed to recreate meaningful participation in practice (7, 27, 38). In many Global South settings, however, authentic clinical work is abundant and attempts to solve local preparedness challenges by implementing Global North solutions would risk missing out on the potential of this rich training context (5). The challenge is not creating participation but legitimising it. This distinction matters because it suggests that some Global South contexts may already possess conditions that align closely with longstanding educational ideals regarding practice-based learning (21).

Rather than viewing the Global South primarily as a site for adaptation, we argue that it should also be viewed as a source of educational innovation. The question is not only how Global South institutions can contextualise imported educational models, but also what Global North institutions might learn from settings where authentic participation remains possible at scale. If participation is legitimised through supportive clinical cultures, meaningful responsibility and assessment systems that value readiness for practice, Global South institutions may be uniquely positioned to produce graduates with exceptional adaptability, resilience and practical know-how. In this respect, legitimacy may represent not merely a local solution to a local problem, but an important contribution to global conversations about how best to prepare future doctors.

## Data Availability

The original contributions presented in the study are included in the article/Supplementary Material, further inquiries can be directed to the corresponding author.
